# Zfp238 Regulates the Thermogenic Program in Cooperation with Foxo1

**DOI:** 10.1016/j.isci.2019.01.005

**Published:** 2019-01-07

**Authors:** Motoko Kita, Jun Nakae, Yoshinaga Kawano, Hiroshi Asahara, Hiroshi Takemori, Haruo Okado, Hiroshi Itoh

**Affiliations:** 1 Navigation Medicine of Kidney and Metabolism, Division of Endocrinology, Metabolism, and Nephrology, Department of Internal Medicine, Keio University School of Medicine, Tokyo 160-8582, Japan; 2 Department of Physiology, International University of Health and Welfare School of Medicine, Narita 286-8686, Japan; 3 Department of Systems BioMedicine, Tokyo Medical and Dental University, Tokyo 113-8519, Japan; 4 Department of Chemistry and Biomolecular Science, Faculty of Engineering, Gifu University, Gifu 501-1193, Japan; 5 Department of Brain Development and Neural Regeneration, Tokyo Metropolitan Institute of Medical Science, Setagaya, Tokyo 156-0057, Japan

**Keywords:** Molecular Interaction, Molecular Mechanism of Behavior, Diabetology, Specialized Functions of Cells

## Abstract

Obesity has become an explicit public health concern because of its relevance to metabolic syndrome. Evidence points to the significance of beige adipocytes in regulating energy expenditure. Here, using yeast two-hybrid screening, we show that Zfp238 is a Foxo1 co-repressor and that adipose-tissue-specific ablation of Zfp238 (*Adipo-Zfp238KO*) in mice leads to obesity, decreased energy expenditure, and insulin resistance under normal chow diet. *Adipo-Zfp238KO* inhibits induction of *Ucp1* expression in subcutaneous adipose tissue upon cold exposure or CL316243, but not in brown adipose tissue. Furthermore, knockdown of Zfp238 in 3T3-L1 cells decreases *Ucp1* expression in response to cool incubation or forskolin significantly compared with control cells. In contrast, overexpression of Zfp238 in 3T3-L1 cells significantly increases *Ucp1* expression in response to forskolin. Finally, double knockdown of both Zfp238 and Foxo1 normalizes *Ucp1* induction. These data suggest that Zfp238 in adipose tissue regulates the thermogenic program in cooperation with Foxo1.

## Highlights

• Zfp238 is a Foxo1 co-repressor
• Zfp238 deficiency in adipocyte leads to obesity and decreased energy expenditure
• Knockdown of Zfp238 in 3T3-L1 cells decreases Ucp1 induction
• Double knockdown of both Zfp238 and Foxo1 normalizes Ucp1 induction

## Graphical Abstract

**Figure:**
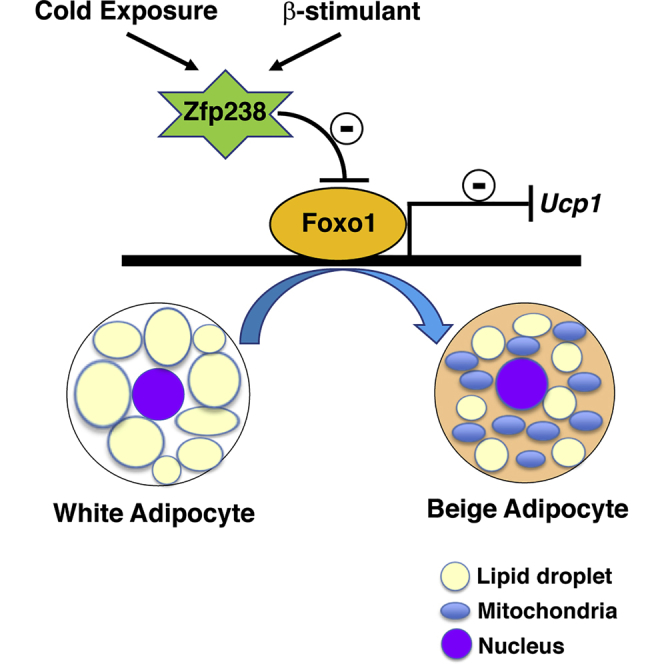

## Introduction

Obesity is an important risk factor for cardiovascular and kidney diseases, diabetes, some cancers, and musculoskeletal disorders (NCD Risk Factor Collaboration (NCD-RisC), 2016). Therefore, it is important to understand how obesity arises and how it can be prevented and treated. Obesity results from energy imbalance and can develop when the energy intake exceeds the energy expenditure (Rosen and Spiegelman, 2006). Adaptive thermogenesis is defined as heat production in response to cold exposure or overfeeding, protecting the organism from cold, or regulating energy balance after changes in diet. Brown adipose tissue (BAT) and skeletal muscle are the two major organs involved in adaptive thermogenesis (Cannon et al., 1998). Rodents have prominent brown fat depots, whereas larger mammals including humans do not, although there may be brown adipocytes dispersed among white adipose tissue (WAT) (Rosen and Spiegelman, 2006).

Recent studies have demonstrated that chronic cold exposure in adults facilitates the accumulation of F 18 fludeoxyglucose positron emission tomography-positive BAT even in people who previously lacked detectable BAT before cold exposure, presumably because of the emergence of new thermogenic adipocytes (Lee et al., 2014, van der Lans et al., 2013, Yoneshiro et al., 2013). Furthermore, recent studies demonstrate that mammals have at least two types of thermogenic adipocytes, the classical brown adipocytes and inducible, termed beige (or brite), adipocytes (Wu et al., 2012). Beige adipocytes emerge postnatally from WAT and are highly induced by various environmental stimuli, including chronic cold exposure, exercise, treatment with β3-agonist, and with peroxisome proliferator-activated receptor-γ (PPARγ) activity (Kajimura et al., 2015).

Forkhead box-containing protein O (Foxo) 1 is a key transcription factor in insulin and glucose metabolism that is phosphorylated, subsequently exported to the cytoplasm, and inhibited by insulin/insulin growth factor 1in a phosphatidylinositol 3-kinase-dependent manner. Foxo1 plays an important role in mediating insulin action in several insulin-responsive tissues (Nakae et al., 2008b). Haploinsufficiency of Foxo1 restores the size of white adipocytes under high-fat diet (Kim et al., 2009, Nakae et al., 2003). Furthermore, overexpression of transactivation-defective Foxo1 in BAT increases O_2_ consumption (Nakae et al., 2008a). Therefore, Foxo1 can be an attractive target for improving energy homeostasis in adipose tissue. However, the physiological role of Foxo1 in beige adipocytes is not known.

In the present study, using yeast two-hybrid screen of a mouse 3T3-L1 cDNA library, we identified Zfp238 (also known as Rp58) as a Foxo1-binding protein (Nakae et al., 2012). We demonstrated that Zfp238 inhibits Foxo1 transcriptional activity and that adipose-tissue-specific *Zfp238* knockout mice (*Adipo-Zfp238KO*) show obesity, decreased whole-body O_2_ consumption, and decreased expression of *Ucp1* stimulated with cold exposure or β3 agonist in subcutaneous adipose tissue. Furthermore, knockdown of Zfp238 in 3T3-L1 cells stimulated with a cool environment or forskolin (FSK) abolished induction of *Ucp1* expression, but double knockdown of both Zfp238 and Foxo1 rescued it, indicating that cooperation between Zfp238 and Foxo1 plays an important role in the thermogenic program in adipose tissue.

## Results

### Identification of Zfp238 as a Foxo1-Binding Protein

Previously, to identify Foxo1-interacting proteins, we performed yeast two-hybrid screen, using a GAL4-Foxo1 fragment (amino acids 1–154) as bait and a mouse 3T3-L1 cDNA library as prey. We selected 17 clones by the criteria described in our previous report (Nakae et al., 2012). Among them, we identified Zfp238, a zinc finger-type transcription factor, as a Foxo1-binding protein.

**Figure 1:**
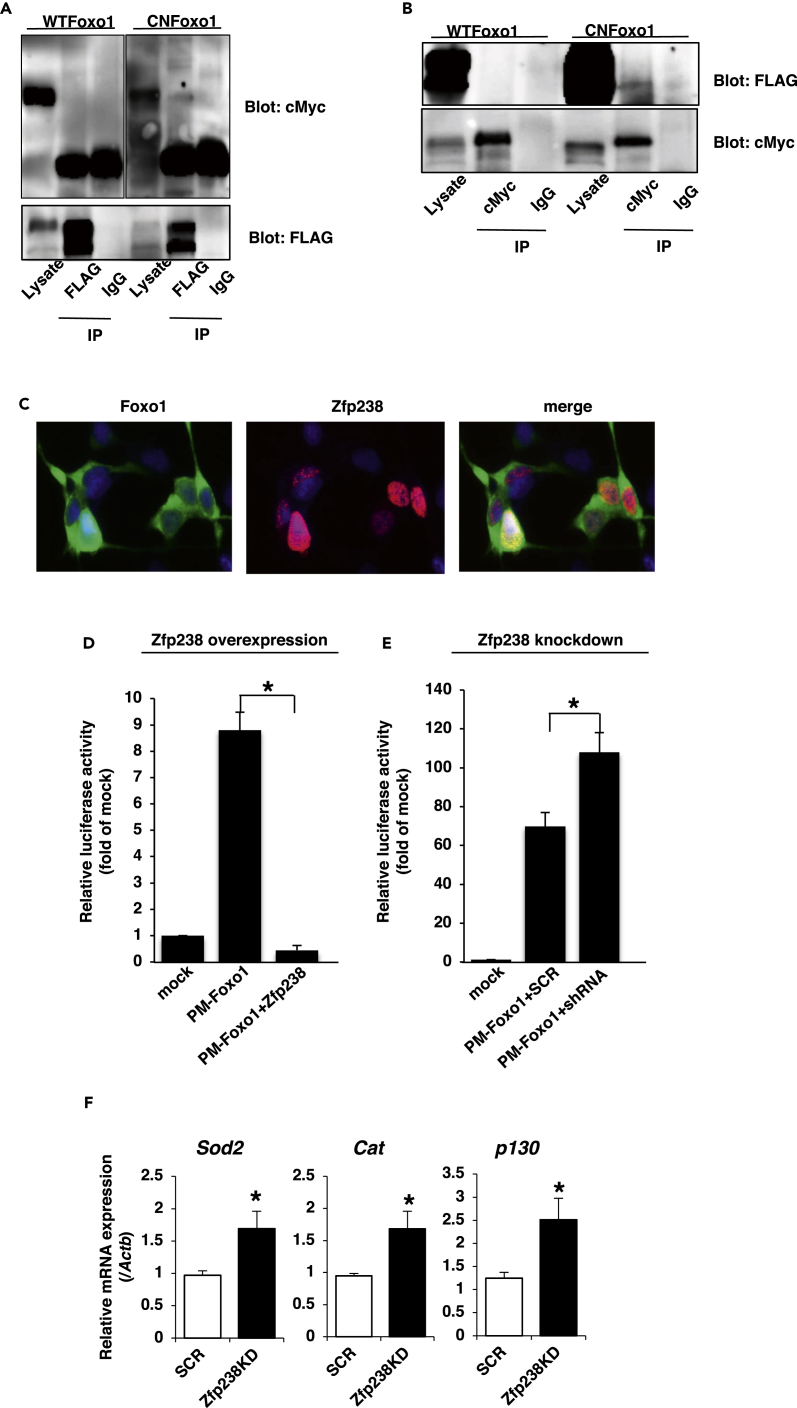
Zfp238 Interacts with Foxo1 and Inhibits Its Activity (A and B) Interaction between exogenous Zfp238 and Foxo1. HEK293 cells were co-transfected with cMyc-tagged WTFoxo1 or CNFoxo1 and FLAG-tagged Zfp238 and cultured in the presence of serum. At 48 h after transfection, cells were harvested and lysates were immunoprecipitated (IP) with anti-FLAG (A) or anti-cMyc (B) or normal mouse IgG and blotted with anti-cMyc (A) or anti-FLAG (B). (C) Immunofluorescence of transfected cMyc-WTFoxo1 and FLAG-Zfp238 in HEK293 cells. (D) Overexpression of Zfp238 inhibits Foxo1-induced 5XGAL4-luciferase activity. At 36 h after co-transfection with pTAL-5XGAL4, phRL-SV40, PM-WTFoxo1, and FLAG-Zfp238 or p3xFlag-CMV empty vector, HEK293 cells were harvested and luciferase activity was measured. An asterisk indicates a statistically significant difference between luciferase activity in the absence and presence of FLAG-Zfp238 (*p < 0.05 by one-way ANOVA). Data represent the mean ± SEM from three independent experiments. (E) Knockdown of Zfp238 increases Foxo1-induced 5XGAL4-luciferase activity. An asterisk indicates a statistically significant difference between luciferase activity in the absence and presence of shRNA-6 Zfp238 (*p < 0.05 by one-way ANOVA). Data represent the mean ± SEM from three independent experiments. (F) Knockdown of Zfp238 induces expression of Foxo1-target genes in differentiated 3T3-L1 cells in the absence of serum. At 10–12 days after induction of differentiation of 3T3-L1 cells infected with pSIREN-RetroQ encoding shRNA-6 Zfp238 or shRNA-SCR, cells were harvested and real-time PCR was performed. An asterisk indicates a statistically significant difference (*p < 0.05 by one-way ANOVA). Data represent the mean ± SEM from three independent experiments.

To confirm the interaction between Foxo1 and Zfp238, we co-transfected HEK293 cells with cMyc-tagged wild-type Foxo1 (WTFoxo1) or constitutively nuclear Foxo1 (CNFoxo1) (Nakae et al., 2006) and FLAG-tagged Zfp238 (Yokoyama et al., 2009) and performed reciprocal immunoprecipitation/immunoblotting experiments in the presence of serum using anti-cMyc and anti-FLAG antibodies. These experiments showed that Zfp238 interacted with CNFoxo1, but not with WTFoxo1 (Figures 1A and 1B). To investigate the subcellular localization of WTFoxo1 and Zfp238, we performed immunofluorescence using HEK293 co-transfected with cMyc-tagged WTFoxo1 and FLAG-tagged Zfp238 in the presence of serum. Immunofluorescence showed that WTFoxo1 was localized mainly in the cytosol, but Zfp238 was localized in the nucleus (Figure 1C). These data suggest that Zfp238 may interact with Foxo1 mainly in the nucleus.

### Zfp238 Inhibits Transcriptional Activity of Foxo1

Transcriptional activity of Foxo1 is mainly inhibited by Akt-mediated phosphorylation and export from nucleus to cytosol. Because Zfp238 is mainly in the nucleus, we performed a transactivation assay using HEK293 cells co-transfected with the GAL4-Foxo1 fusion protein, FLAG-tagged Zfp238, and 5XGAL4-luciferase vectors. Overexpression of Zfp238 inhibited Foxo1-induced transactivation significantly (Figure 1D). In contrast, knockdown of endogenous Zfp238 using short hairpin RNA (shRNA) significantly increased Foxo1-induced transcriptional activity (Figure 1E).

To investigate the effects of Zfp238 on endogenous Foxo1 transcriptional activity, we knocked down endogenous Zfp238 in the preadipocyte cell line 3T3-L1 using a retrovirus encoding the shRNA of Zfp238 (Figure S1A). Real-time PCR revealed that knockdown of Zfp238 in mature 3T3-L1 had no effect on the expression levels of endogenous Foxo1 target genes in the presence of serum (Figure S1B). However, knockdown of Zfp238 significantly increased the expression levels of endogenous Foxo1 target genes in the absence of serum (Figure 1F). These data suggest that Zfp238 inhibits transcriptional activity of Foxo1.

It is known that one of the mechanisms by which the transcriptional activity of Foxo1 is inhibited is its phosphorylation and export from the nucleus (Nakae et al., 1999, Nakae et al., 2000). However, ectopic expression of FLAG-tagged Zfp238 in HEK293 cells significantly increased nuclear localization of Foxo1 (Figure S1C). These data indicate that Zfp238 inhibits transcriptional activity of Foxo1 in the nucleus, but not by increasing cytosolic localization.

### Zfp238 Is Expressed in Adipose Tissue

**Figure 2:**
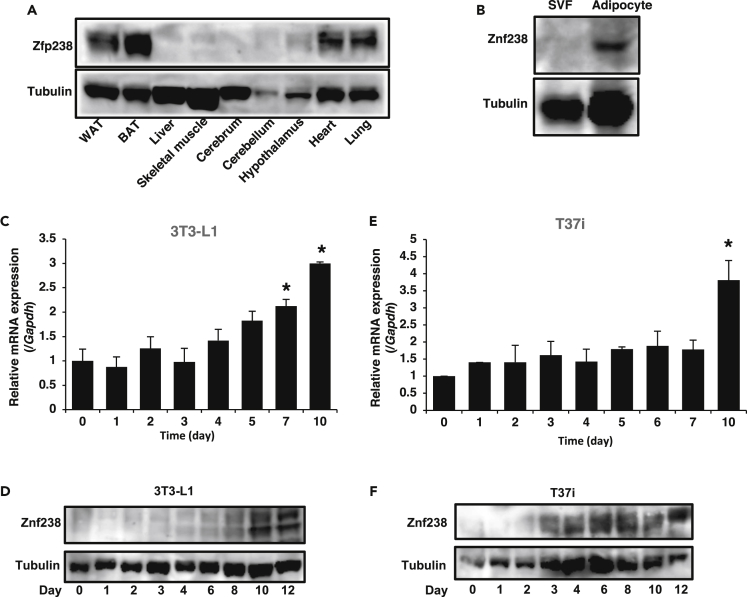
Zfp238 Is Expressed in Adipose Tissues (A) Western blot of Zfp238 in various tissues. Protein lysates from the indicated tissues were subjected to western blot with anti-Zfp238 and anti-tubulin antibodies. (B) Western blot of Zfp238 using the stromal vascular and adipocytes fractions of fractionated WAT. (C and D) Real-time PCR of Zfp238 from 3T3-L1 (C) and T37i cells (D) during differentiation. An asterisk indicates a statistically significant difference between day 0 and day 7 or 10 (*p < 0.05 by one-way ANOVA). Data represent the mean ± SEM from three independent experiments. (E and F) Western blot of Zfp238 protein from 3T3-L1 (E) and T37i (F) cells during differentiation. Lysates from cells on the indicated day after induction of differentiation were subjected to western blot.

Zfp238 is reported to have an essential role in normal brain development and skeletal myogenesis (Okado et al., 2009, Yokoyama et al., 2009). However, studies in adipose tissue are lacking, so we performed expression profiling of Zfp238. Western blot revealed Zfp238 expression in WAT and BAT other than heart and lung (Figure 2A). Fractionation of WAT showed that Zfp238 is expressed mainly in the adipocyte fraction (Figure 2B). Zfp238 was present in both 3T3-L1 cells and brown adipocyte T37i cells in a differentiation-dependent manner (Figures 2C–2F). Oil red O staining showed that knockdown of Zfp238 in 3T3-L1 cells did not affect adipocyte differentiation (Figure S2A). However, real-time PCR results indicated that knockdown of Zfp238 significantly decreased the expression levels of adipocyte-specific genes, including *Adipoq*, *Cebpa*, and *Ppargc1a*, in differentiated 3T3-L1 cells (Figure S2B). Furthermore, overexpression of Zfp238 significantly increased *Adipoq*, *Slc2a4*, and *Fasn* in differentiated 3T3-L1 cells (Figures S2C and S2D). These results suggest that Zfp238 is expressed in adipocytes and may have a functional role in adipose tissues *in vivo*.

### *Adipo-Zfp238 KO* Mice Develop Obesity and Insulin Resistance

**Figure 3:**
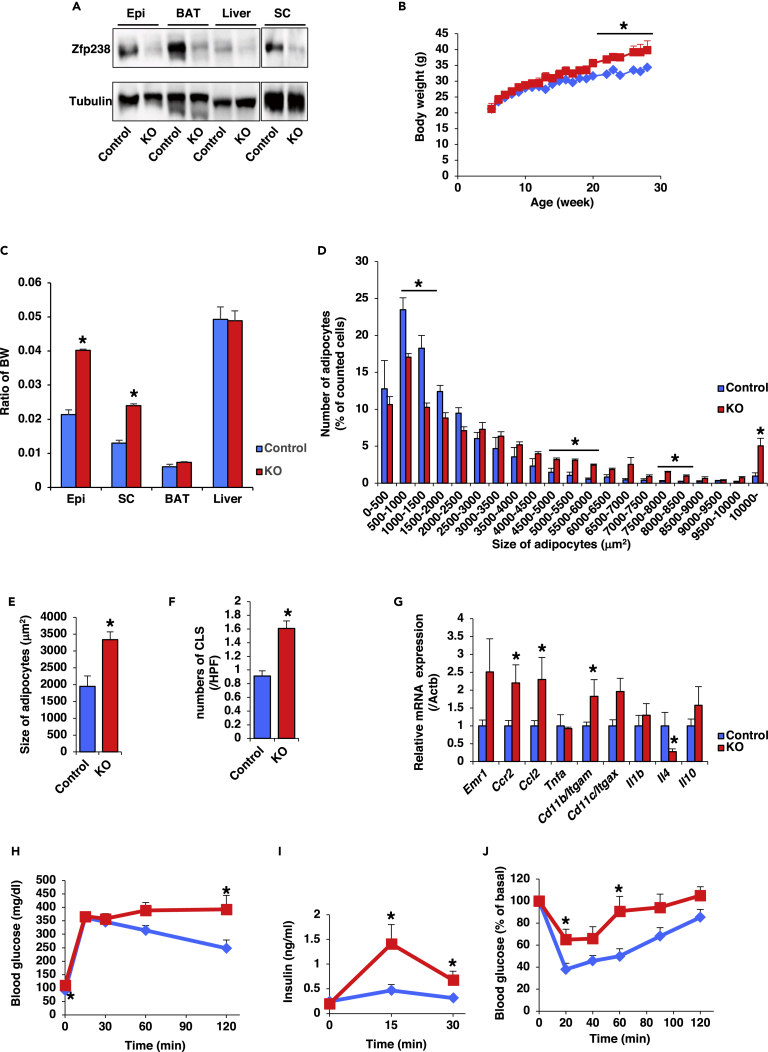
*Adipo-Zfp238 KO* Mice Develop Obesity and Insulin Resistance (A) Western blot of Zfp238 protein in epididymal fat (Epi), BAT, liver, and subcutaneous fat (SC) from control and *Adipo-Zfp238 KO* mice. (B) Body weight of control (blue diamond) and *Adipo-Zfp238 KO* (red square) animals fed with NCD (n = 20–24). Data are means ± SEM. *p < 0.05 by one-way ANOVA. (C) Tissue weights of epididymal fat (Epi), subcutaneous fat (SC), BAT, and liver from 20- to 24-week-old control (blue bar) and *Adipo-Zfp238 KO* (red bar) mice under NCD. Data are the ratio of body weight and expressed as means ± SEM (n = 20–24). *p < 0.05 by one-way ANOVA. (D) Histogram of adipocyte size and number of epididymal fat from control (blue bar) and *Adipo-Zfp238 KO* (red bar) mice fed with NCD at the age of 20–24 weeks (n = 8–10). Data represent percentage of total counted cells and means ± SEM. *p < 0.05 by one-way ANOVA. (E) Mean size of adipocytes of epididymal fat (n = 8–10). Data represent each adipocyte area (μm^2^) and means ± SEM. *p < 0.05 by one-way ANOVA. (F) The number of crown-like structures (CLSs) in epididymal fat of mice at the age of 20–24 weeks (n = 8–10). Data represent the numbers of CLSs in 1 high power field (HPF) (100 X) and means ± SEM. *p < 0.05 by one-way ANOVA. (G) Normalized gene expression of immune-cell-related and cytokine genes in epididymal fat in mice at the age of 20–24 weeks (n = 8–10). Data are the ratio of control in each gene and means ± SEM. *p < 0.05 by one-way ANOVA. (H) IPGTT of control (blue diamond) and *Adipo-Zfp238 KO* (red square) animals fed with NCD at the age of 20–22 weeks (n = 10–12). Data are means ± SEM. (I) Insulin secretion of control and *Adipo-Zfp238 KO* mice during IPGTT. Data are means ± SEM. *p < 0.05 by two-way ANOVA with Fisher's test. (J) Insulin tolerance test of control and *Adipo-Zfp238 KO* mice fed with NCD at the age of 20–22 weeks (n = 10–12). Data are means ± SEM. *p < 0.05 by two-way ANOVA with Fisher's test.

To investigate the functional role of Zfp238 in adipose tissues, we generated adipose-tissue-specific *Zfp238* knockout (*Adipo-Zfp238 KO*) mice by crossing conditional *Zfp238* allele (Ohtaka-Maruyama et al., 2013) with *Adiponectin-Cre* mice (Eguchi et al., 2011). Western blot revealed a marked reduction of Zfp238 protein in epididymal fat, subcutaneous fat, and BAT from *Adipo-Zfp238 KO* mice (Figure 3A). Zfp238 protein expression in liver from control mice was lower than in adipose tissues, so comparison of Zfp238 expression level between control and *Adipo-Zfp238 KO* animals was difficult (Figure 3A).

*Adipo-Zfp238 KO* mice demonstrated increased weight gain on a normal chow diet (NCD) after 20 weeks of age (Figure 3B). Analysis of tissue weights revealed that epididymal and subcutaneous fat weights from *Adipo-Zfp238 KO* animals significantly increased compared with controls, with no differences in BAT and liver (Figure 3C). Adipocytes in epididymal fat from *Adipo-Zfp238 KO* mice were significantly larger than those from controls (Figure S3A). Quantitative assessment confirmed a 70% increase in mean adipocyte area in the epididymal fat from *Adipo-Zfp238 KO* mice (Figures 3D and 3E). Furthermore, mean adipocyte area of subcutaneous fat from *Adipo-Zfp238 KO* mice also tended to be larger than from control mice (Figures S3B and S3C). mRNA expression in epididymal and subcutaneous fats of *Adipo-Zfp238 KO* animals did not differ for general markers of adipose-tissue-specific genes except *Glut 4* (Figures S3D and S3E).

Because obesity leads to chronic inflammation in WAT and to insulin resistance (Olefsky and Glass, 2010), we examined infiltration of inflammatory macrophages in epididymal fat. A crown-like structure (CLS) in adipose tissue is the accumulation of immune cells around dead adipocytes (Murano et al., 2008). We found that the number of F4/80^+^ CLSs per field in epididymal fat was significantly higher in *Adipo-Zfp238 KO* animals than in controls (Figure 3F). Consistent with these findings, the expression levels of C-C chemokine receptor type 2 (*Ccr2*), chemokine (C-C motif) ligand 2 (*Ccl2*), and *Cd11b* were significantly increased and expression level of *Il4*, an anti-inflammatory cytokine, was significantly decreased in epididymal fat from *Adipo-Zfp238 KO* compared with from control animals (Figure 3G). However, the expression levels of these genes in subcutaneous fat from *Adipo-Zfp238 KO* were similar to those in control animals (Figure S3F). These data indicate that chronic inflammation of epididymal fat from *Adipo-Zfp238 KO* mice was increased.

We next assessed glucose homeostasis in *Adipo-Zfp238 KO* mice. The intraperitoneal glucose tolerance test (IPGTT) revealed that *Adipo-Zfp238 KO* mice had glucose intolerance (Figure 3H). Moreover, insulin secretion of *Adipo-Zfp238 KO* mice during IPGTT was significantly increased compared with controls (Figure 3I). Furthermore, the insulin tolerance test demonstrated that *Adipo-Zfp238 KO* mice had significant insulin resistance (Figure 3J). These data suggest that deletion of Zfp238 in adipose tissues deteriorates insulin sensitivity.

### Zfp238 Regulates Whole-Body Energy Expenditure

**Figure 4:**
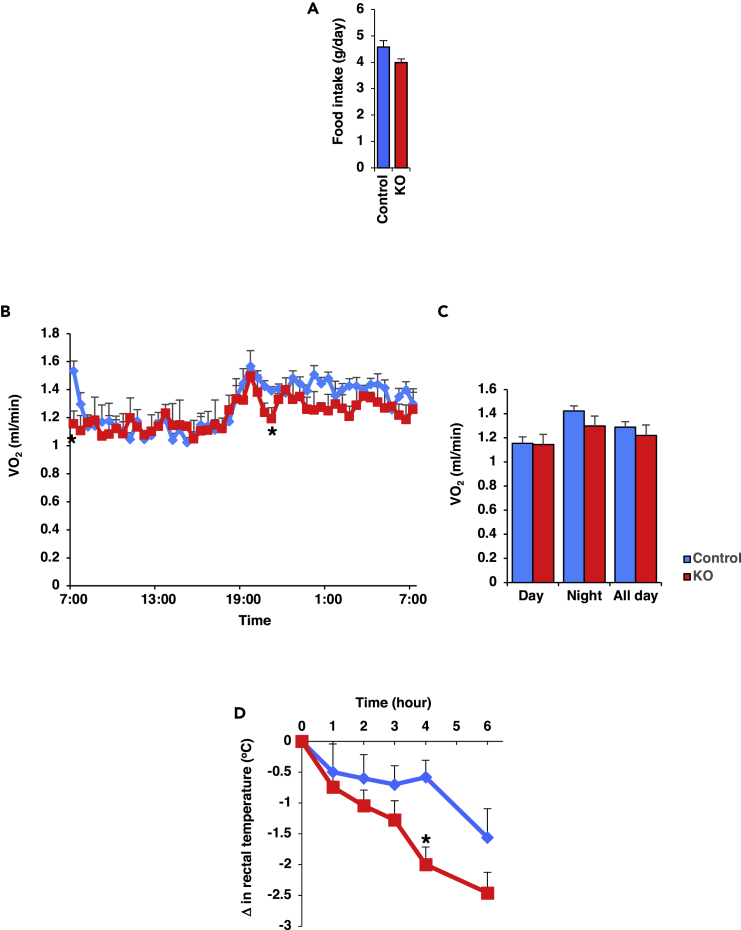
Zfp238 Regulates Whole-Body Energy Expenditure (A) Food intake of 14- to 16-week-old control and *Adipo-Zfp238 KO* mice. Data represent mean ± SEM of food intake for 4 days. (B) The oxygen consumption of control (blue diamond) and *Adipo-Zfp238 KO* (red square) animals fed with NCD at the age of 14–16 weeks (n = 7–8). Data are means ± SEM. *p < 0.05 by two-way ANOVA with Fisher's test. (C) Means ± SEM of the oxygen consumption during daytime, nighttime, and all day. *p < 0.05 by one-way ANOVA. (D) Changes in rectal temperature of 16-week-old control (blue diamond) and *Adipo-Zfp238 KO* (red square) mice after cold exposure (n = 7). Data are means ± SEM. *p < 0.05 by two-way ANOVA with Fisher's test.

To investigate the mechanism of how body weight was increased, we first measured food intake, which did not differ between control and *Adipo-Zfp238 KO* animals (Figure 4A). Given the preferential effect of *Zfp238* deletion on body weight, we assessed the physiological effect of this ablation, using O_2_ consumption. We measured O_2_ consumption using indirect calorimetry. Oxygen consumption levels are affected by body weight (Tschop et al., 2011). Therefore, we also estimated oxygen consumption data without normalization by body weight. The oxygen consumption of *Adipo-Zfp238 KO* without normalization by body weight also tended to be lower than control (Figures 4B and 4C). The respiratory quotient of *Adipo-Zfp238 KO* was similar to controls (data not shown). Furthermore, the decline in rectal temperature of *Adipo-Zfp238 KO* animals was significantly steeper compared with controls at 4°C (Figure 4D). These data suggest that ablation of Zfp238 in adipose tissues decreased energy expenditure.

### Ablation of *Zfp238* Affects the Thermogenic Gene Program

In contrast to WAT, which stores excess energy, BAT dissipates energy and produces heat as a defense against hypothermia and obesity in mammals. Therefore, microscopic morphology of BAT was examined in control and *Adipo-Zfp238 KO* mice. Although brown adipocytes in control had multilocular lipid droplets, which were typical brown adipocytes, brown adipocytes in *Adipo-Zfp238 KO* animals had large lipid droplets (Figure S4A). However, real-time PCR revealed that gene expression levels of BAT-specific thermogenic genes except *Cidea* in BAT of *Adipo-Zfp238 KO* animals were similar to controls under room temperature (Figure S4B).

**Figure 5:**
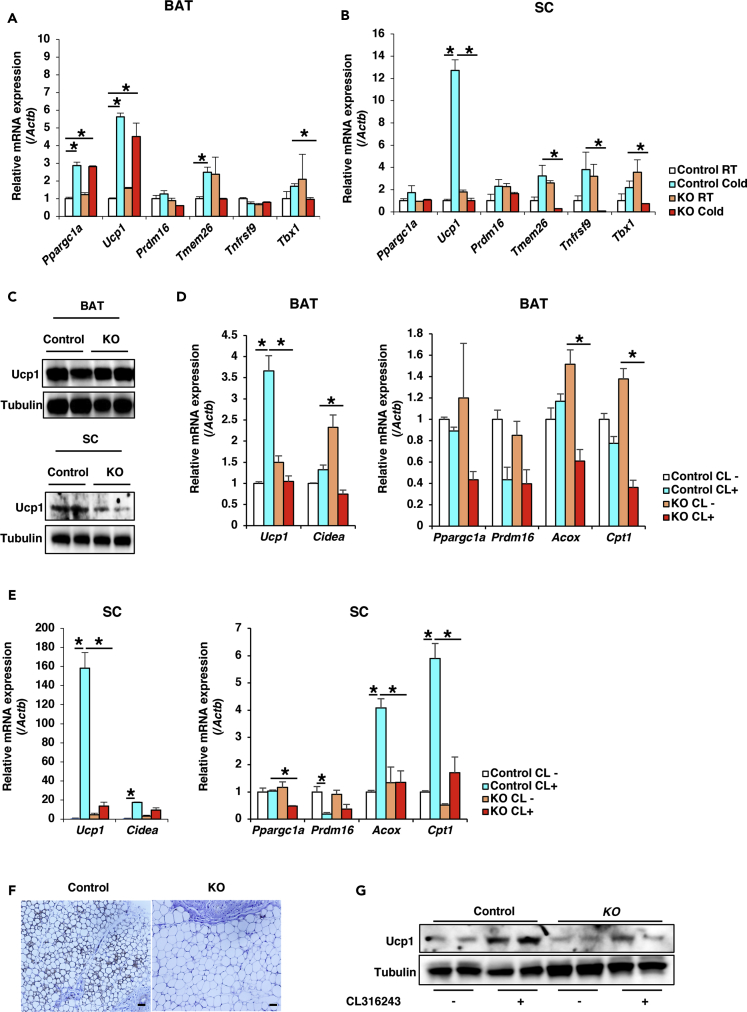
Ablation of *Zfp238* Affects the Thermogenic Gene Program (A and B) Gene expression levels of beige-adipocyte-related genes in BAT (A) and SC (B) from 16-week-old control and *Adipo-Zfp238 KO* mice at room temperature (RT) and under cold exposure (n = 4–6). Data represent the ratio of control at RT and means ± SEM. *p < 0.05 by one-way ANOVA. (C) Representative western blot of Ucp1 protein expression in BAT and SC from control and *Adipo-Zfp238 KO* mice with cold exposure. (D and E) Thermogenic gene expression in BAT (D) and SC (E) from 16-week-old control and *Adipo-Zfp238 KO* mice stimulated with CL for 6 days (n = 6). Data represent the ratio of control at basal state and means ± SEM. *p < 0.05 by one-way ANOVA. (F) Representative images from UCP1 staining on section of SC from 16-week-old control and *Adipo-Zfp238 KO* mice after stimulation with CL (scale bar, 20 μm). (G) Representative western blot of Ucp1 protein expression in SC from control and *Adipo-Zfp238 KO* mice at stimulation with CL.

Beige adipocytes are sporadically localized in subcutaneous WAT and emerge under certain external cues, such as chronic cold exposure and exercise. Therefore, we first examined gene expression in subcutaneous adipose tissue of control and *Adipo-Zfp238 KO* animals at room temperature. Real-time PCR revealed that some thermogenic genes related to beige adipocytes, including *Ucp1*, *Cidea*, *Acox1*, *Esrra*, *Nrf1*, and *Ppara*, were significantly upregulated in *Adipo-Zfp238 KO* mice compared with control (Figure S4C). However, because BAT and subcutaneous WAT are prone to inducing a thermogenic gene program, we examined the effect of cold exposure on the expression levels of thermogenic genes. Of interest, *Zfp238* gene expression level in subcutaneous WAT under cold exposure was significantly increased compared with levels at room temperature. In contrast, Zfp238 gene expression level in BAT at cold exposure was significantly decreased compared with room temperature (Figure S4D). *Ppargc1a* and *Ucp1* gene expression levels in BAT from *Adipo-Zfp238 KO* mice were induced by cold exposure similarly to controls (Figure 5A). In contrast, *Ucp1* expression in subcutaneous WAT from *Adipo-Zfp238 KO* animals was significantly reduced compared with controls. Furthermore, *Adipo-Zfp238 KO* beige adipocyte-specific gene expression, including of *Tmem26*, *Tnfrsf9*, and *Tbx1*, was also significantly decreased compared with controls (Figure 5B). Consistent with these data, although Ucp1 protein expression level in BAT from *Adipo-Zfp238 KO* animals was similar to control, Ucp1 protein expression level in subcutaneous WAT from *Adipo-Zfp238 KO* mice was significantly decreased compared with controls (Figure 5C).

We also examined the effects of another stimulus for inducing the thermogenic gene program: treatment with the β3-adrenergic agonist CL316243 (referred to here as CL). CL treatment significantly increased *Ucp1* expression levels in BAT from control, but not from *Adipo-Zfp238 KO* mice (Figure 5D). Furthermore, CL treatment significantly increased *Ucp1*, *Cidea*, *Acox*, and *Cpt1* in subcutaneous WAT from control, but not from the KOs (Figure 5E). Moreover, immunohistochemistry with Ucp1 antibody revealed that CL-treatment-induced Ucp1 protein expression in subcutaneous WAT from control was abolished in *Adipo-Zfp238 KO* animals (Figure 5F). Consistent with these data, western blot showed that CL-treatment-induced Ucp1 protein expression level in subcutaneous WAT of KOs was significantly decreased compared with controls (Figure 5G). These data indicate that ablation of *Zfp238* in adipose tissues declines under the thermogenic gene program.

### Zfp238 Regulates *Ucp1* Expression by Inhibition of Foxo1

**Figure 6:**
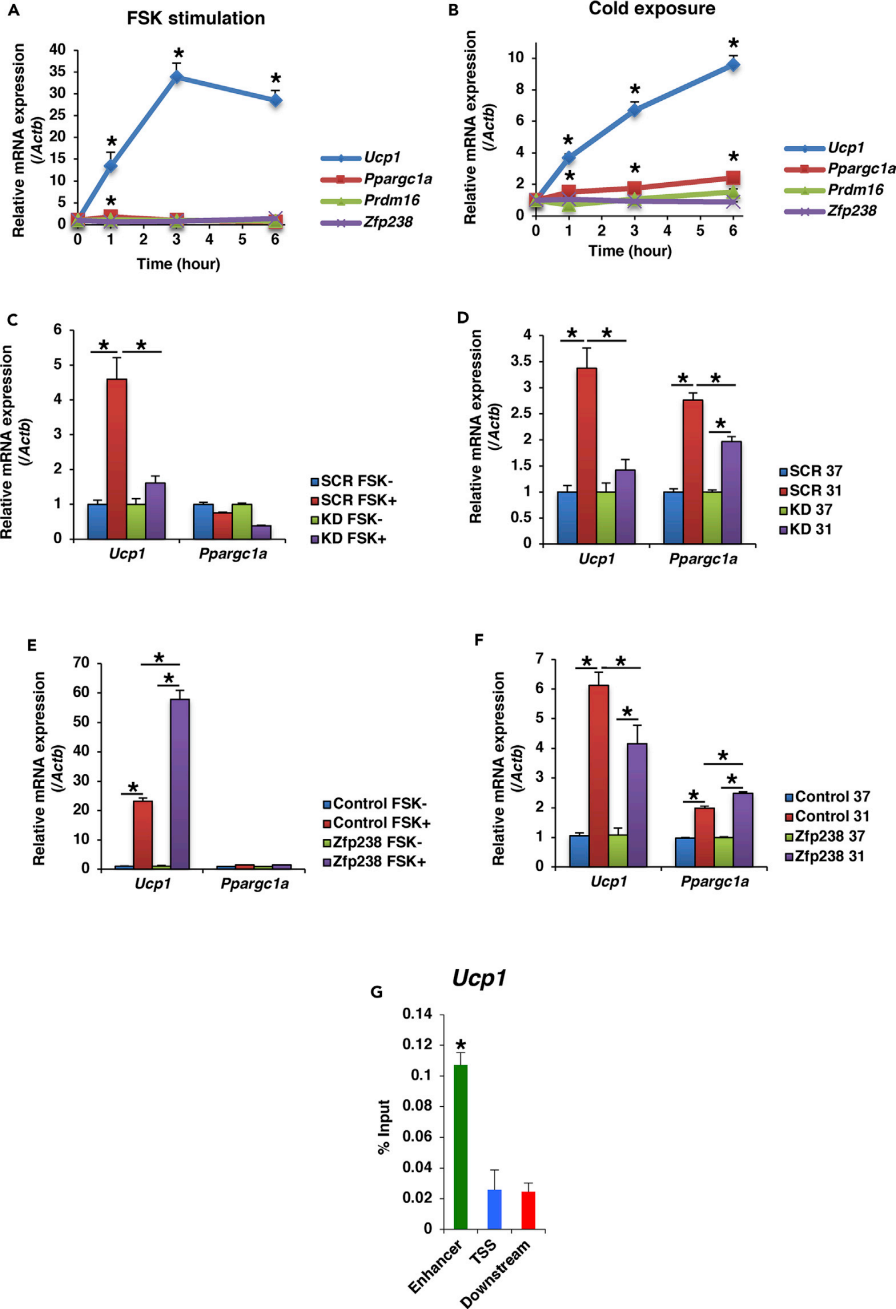
Zfp238 Regulates *Ucp1* Expression in 3T3-L1 Cells (A) Time course of gene expression in differentiated 3T3-L1 cells stimulated with forskolin (FSK). (B) Time course of gene expression in differentiated 3T3-L1 cells incubated at 31°C. Experiments were performed three times. Data at each time point represent the ratio of gene expression level at basal state and means ± SEM. *p < 0.05 by two-way ANOVA with Fisher's test. (C and D) Effects of knockdown of Zfp238 in differentiated 3T3-L1 cells on *Ucp1* and *Ppargc1a* expression induced by FSK (C) or 6-h incubation at 31°C (D). Experiments were performed three times. Data represent the ratio of gene expression level in 3T3-L1 cells infected with retroviruses encoding shRNA-SCR at basal state and means ± SEM. *p < 0.05 by one-way ANOVA. (E and F) Effects of overexpression of Zfp238 on *Ucp1* and *Ppargc1a* expression in differentiated 3T3-L1 cells. Experiments were done three times. Data represent the ratio of gene expression level in 3T3-L1 cells infected with retroviruses encoding FLAG empty vector (control) at basal state and means ± SEM. *p < 0.05 by one-way ANOVA. (G) Normalization of data of chromatin immunoprecipitation assay. Experiments were performed three times. Data represent the percentage of density of input performed by real-time PCR and means ± SEM. *p < 0.05 by one-way ANOVA.

We next examined the effects of Zfp238 on the thermogenic gene program at the cellular level. Stimulation with 10 μM FSK significantly increased the *Ucp1* expression in 3T3-L1 cells (Figure 6A). Furthermore, incubation of 3T3-L1 cells at 31°C significantly increased *Ucp1* and *Ppargc1a* expression (Figure 6B). Based on these results, we examined *Ucp1* and *Ppargc1a* expression in response to FSK stimulation or incubation at 31°C using Zfp238-knockdown or Zfp238-overexpressed 3T3-L1 cells. Stimulation with FSK significantly increased *Ucp1* expression in SCR-3T3-L1 cells, but not in Zfp238-knockdown cells (Figure 6C). Furthermore, incubation at 31°C significantly increased *Ucp1* expression in SCR-3T3-L1 cells, but not in Zfp238-knockdown cells, although *Ppargc1a* expression was significantly induced in both cell lines (Figure 6D). In contrast, *Ucp1* expression in Zfp238-overexpressed 3T3-L1 cells stimulated by FSK was significantly increased compared with mock-transfected cells, although *Ucp1* and *Ppargc1a* expression levels in Zfp238-overexpressed 3T3-L1 cells incubated at 31°C were similar to those in mock-transfected cells (Figures 6E and 6F). Furthermore, chromatin immunoprecipitation assay demonstrated that Zfp238 bound to the enhancer region of the *Ucp1* promoter, but not to the transcriptional start site (TSS) or a region 5 kb downstream of the TSS (Iida et al., 2015) (Figure 6G). These data suggest that Zfp238 regulates *Ucp1* expression.

**Figure 7:**
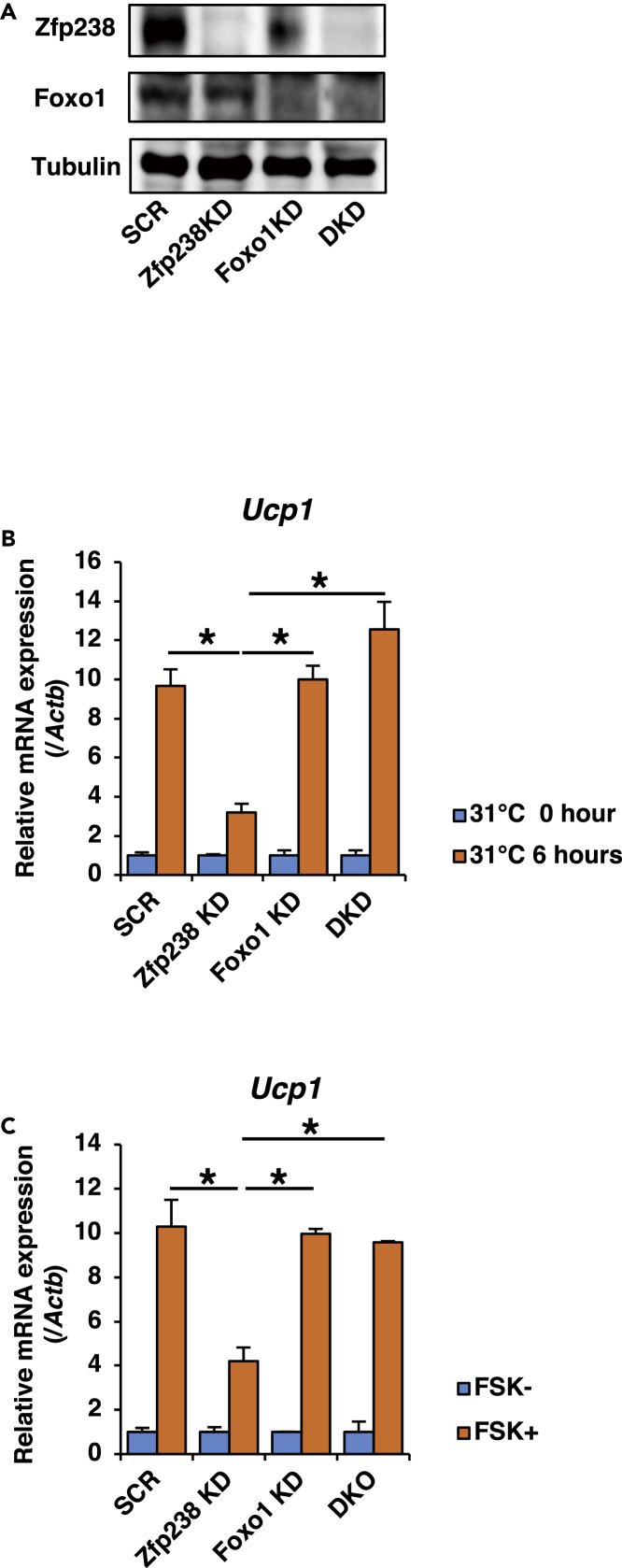
Zfp238 Regulates Ucp1 Expression by Inhibiting Foxo1 (A) Representative western blot of Zfp238 and Foxo1 in differentiated 3T3-L1 cells infected with retroviruses encoding pSINsi-DK II-Zfp238, pSINsi-DK II-SCR, pSINsi-DK II-Foxo1, or double knockdown (DKO) of Zfp238 and Foxo1. (B and C) Effects of double knockdown of Zfp238 and Foxo1 in differentiated 3T3-L1 cells on *Ucp1* expression induced by 31°C (B) or FSK (C). Experiments were performed three times. Data represent the ratio of gene expression level in 3T3-L1 cells at each basal state and means ± SEM. *p < 0.05 by one-way ANOVA.

If Zfp238 binds to Foxo1 and inhibits its transcriptional activity, then ablation of *Zfp238* in adipose tissues would be expected to increase Foxo1 activity (Figure 1F). In turn, if ablation of Zfp238 inhibits *Ucp1* expression by Foxo1 activation, then knockdown of Foxo1 would be expected to normalize *Ucp1* expression. Therefore, we generated Zfp238-and Foxo1-double knockdown 3T3-L1 cells using a retroviral system and examined *Ucp1* expression levels with incubation at 31°C or stimulation with FSK (Figure 7A). *Ucp1* expression level in SCR- and Foxo1-knockdown cells was significantly increased compared with Zfp238-knockdown cells with incubation at 31°C. Double knockdown of both Zfp238 and Foxo1 significantly increased and normalized *Ucp1* expression (Figure 7B). Furthermore, stimulation with FSK significantly increased *Ucp1* expression in SCR- and Foxo1-knockdown cells compared with Zfp238-knockdown cells, and double knockdown of both Zfp238 and Foxo1 normalized *Ucp1* expression (Figure 7C). These data indicate that Zfp238 regulates *Ucp1* expression by the inhibition of Foxo1.

## Discussion

Brown and beige adipocytes expend chemical energy to produce heat and are thus important for regulating body temperature and body weight (Wang and Seale, 2016). Beige adipocytes are induced to develop in WAT in response to environmental cues, including cold exposure, exercise, cachexia, and bariatric surgery (Kajimura et al., 2015). These responses to various environmental stimuli for promoting beige adipocyte biogenesis require a number of transcriptional and epigenetic regulators (Inagaki et al., 2016).

In the present study, we have identified Zfp238 as a Foxo1-binding protein. Zfp238 is a sequence-specific transcriptional repressor, sharing homology with a number of transcriptional regulators at the amino terminus, termed the POZ domain (Aoki et al., 1998). Zfp238 has been identified as a transcriptional repressor of Id1-4 and Ngn2 in the neurogenesis of the central nervous system (Hirai et al., 2012) (Ohtaka-Maruyama et al., 2013) and as a transcriptional repressor of Id2 and Id3 in the myogenic program (Yokoyama et al., 2009). Furthermore, the DNA methyltransferase, Dnmt3a, associates with Zfp238, leading to transcriptionally silent heterochromatin (Fuks et al., 2001). The activity of Foxo1 is regulated by posttranslational modification, such as phosphorylation, acetylation, methylation, and ubiquitination (Nakae et al., 2008b, Yamagata et al., 2008). The recruitment of cofactors, including PGC1a, PRMT, FCoR, and Sin3a, is also important for the regulation of Foxo1 activity (Puigserver et al., 2003, Choi et al., 2012, Nakae et al., 2012, Langlet et al., 2017, van der Vos and Coffer, 2008). Although Zfp238 has an inhibitory effect on gene transcription, little is known about its function as a corepressor for other transcription factors, such as Foxo1.

Zfp238 is expressed in adipose tissues, including WAT and BAT, and especially in mature adipocytes. Although Zfp238 does not affect adipocyte differentiation, it has some effects on the regulation of expression levels of adipocyte-specific genes, such as *Adipoq*, *Slc4a2*, *Fasn*, and *Ppargc1a*, leading to the hypothesis that Zfp238 may have an important physiological role in adipose tissues. Here, we have described a mouse model with specific ablation of *Zfp238* in adipose tissues. *Adipo-Zfp238KO* animals show obesity, decreased whole-body O_2_ consumption, and cold intolerance under NCD.

In the basal state, thermogenic gene expression levels in BAT and subcutaneous adipose tissue of *Adipo-Zfp238KO* animals were not suppressed compared with control mice. Especially, thermogenic gene expression levels in subcutaneous adipose tissue of *Adipo-Zfp238KO* were significantly increased compared with control mice. However, during the acute cold challenge, *Adipo-Zfp238KO* animals were hypothermic. This is mainly caused by the impaired BAT activity rather than browning, which usually takes place after chronic cold challenge. This actually explained the compensatory increased thermogenic genes in subcutaneous adipose tissue. It has been already reported that thermogenic capacity is antagonistically regulated in brown and white subcutaneous adipose tissues (Wu et al., 2014). However, following stimulation with cold or the β3-adrenergic agonist CL, induction of the thermogenic gene expression, especially *Ucp1*, *Cidea*, *Acox*, and *Cpt1*, was abolished especially in subcutaneous adipose tissue of *Adipo-Zfp238KO* animals. In contrast, stimulation with cold exposure did not affect the induction of *Ppargc1a* and *Ucp1* in BAT of the KOs. It has been suggested that white and beige adipocytes can respond to cool temperature, but classic brown adipocytes do not, and that this activation in beige adipocytes is independent of the canonical cyclic AMP (cAMP)/protein kinase A/cAMP response element-binding protein pathway downstream of the β-adrenergic receptors (Ye et al., 2013). Of interest, cold exposure induced *Zfp238* expression only in subcutaneous adipose tissue. In contrast, *Zfp238* expression in BAT was significantly decreased upon cold exposure. Therefore, Zfp238 may have an important physiological role mainly in subcutaneous tissue under cold conditions, although the mechanism by which the differences of cold-induced *Zfp238* expression between these two adipose tissues is still unknown.

*Ucp1* is the gene most affected by ablation of Zfp238 in adipose tissues and 3T3-L1 cells. It mediates the thermogenic function in brown and beige adipocytes, resides in the inner mitochondrial membrane, and dissipates the proton gradient generated by the electron transport chain. This futile cycle of proton pump and leaking reduces the mitochondrial membrane potential, which in turn leads to high levels of substrate oxidation and the generation of heat (Cannon and Nedergaard, 2004, Lowell and Spiegelman, 2000). The main regulator of *Ucp1* expression is the β-adrenergic receptor signaling pathway, including cAMP-dependent protein kinase and members of the mitogen-activated protein kinase family (Collins et al., 2010). Indeed, *Ucp1* expression was acutely induced by stimulation with FSK, which interacts directly with the catalytic subunit of adenylate cyclase, activating the enzyme and raising the intracellular levels of cAMP, and by incubation at a cool temperature (31°C) even at cellular level. Both stimuli induced *Ucp1* expression in SCR-3T3-L1 cells but not in Zfp238-knockdown 3T3-L1 cells. In contrast, overexpression of Zfp238 significantly induced *Ucp1* expression stimulated by FSK, but we saw no enhancement of *Ucp1* expression induced by incubation at 31°C. Independence of the activation of the canonical cAMP/protein kinase A/cAMP response element-binding protein pathway downstream of the β-adrenergic receptors with incubation at cool temperature may explain these differences (Ye et al., 2013). Several transcription factors and co-regulators have been implicated in the transcriptional activation of *Ucp1*, including PPARs, PGC1α, and ATF2 (Collins et al., 2010, Kang et al., 2005). These factors act through an enhancer element located 2.5 kb upstream of the TSS of *Ucp1* gene. However, little is known about the cold-inducible transcription factors that activate *Ucp1* expression in a tissue-specific manner. Recently, Zfp516, which is a cold-inducible transcription factor enriched in BAT that binds to the promoter region of UCP1 and directly interacts with PRDM16, has been reported as a candidate cold-inducible and tissue-specific transcription factor for the activation of thermogenic genes (Dempersmier et al., 2015). Of interest, Zfp238 is also a cold-inducible transcription factor in subcutaneous adipose tissue, but not in epididymal adipose tissue and BAT.

Zfp238 inhibits Foxo1 activity, although further investigation will be needed for clarifying the mechanism by which Zfp238 inhibits Foxo1, so ablation of *Zfp238* in adipose tissues would be expected to increase Foxo1 activity and a double ablation of Zfp238 and Foxo1 to normalize the phenotype. Indeed, the double knockdown of Zfp238 and Foxo1 in 3T3-L1 cells normalized *Ucp1* expression stimulated with incubation at a cool temperature or with FSK. Our previous work demonstrated that overexpression of a transactivation-defective Foxo1 (Δ256Foxo1) in adipose tissues using *aP2* promoter increases O_2_ consumption and *Ucp1* expression in BAT. In contrast, CNFoxo1 suppressed *Ucp1* expression inT37i brown adipocyte, although, at that time, the investigation of gene expression of subcutaneous adipose tissue and the presence of beige adipocytes were not performed (Nakae et al., 2008a). These data suggest that Foxo1 may inhibit *Ucp1* expression in adipose tissue and suppress energy expenditure and raise the possibility that Foxo1 might suppress the development of beige adipocytes. Furthermore, FLAG-tagged Zfp238 can bind to the enhancer region of the *Ucp1* gene, suggesting that Zfp238 is in upstream of Ucp1. However, the *Ucp1* promoter region between the TSS and 3.5 kb upstream has no consensus Zfp238-binding elements ((a/c)acatctg(g/t)(a/c)) (Aoki et al., 1998). In contrast, this promoter region has several consensus Foxo1-binding elements (gtaaa(c/t)a) (Kawano et al., 2012). Therefore, Zfp238 may bind to the *Ucp1* enhancer region through Foxo1 and regulate energy expenditure through inhibition of Foxo1.

Recent findings have shown that the cross talk of brown and beige adipocytes with immune cells is important to thermogenic activation. The pro-inflammatory cytokines secreted by the infiltrating M1 macrophages of obese WAT might interfere with beige adipogenesis (Chiang et al., 2009), whereas non-inflammatory, alternative activated M2 macrophages exert the thermogenic activity and sympathetic tone of BAT and beige adipose tissue (Nguyen et al., 2011). Various immune cell types, including macrophages, eosinophils, ILC2, and T lymphocytes, act inside adipose tissues to govern the thermogenic activation and recruitment of brown and beige adipose tissues (Villarroya et al., 2018). In WAT of *Adipo-Zfp238KO*, expression levels of gene markers of the pro-inflammatory immune cells were significantly increased, but expression level of an anti-inflammatory type 2 cytokine, *Il4*, was significantly decreased. These immune environmental circumstances might also contribute to the inhibition of beiging in WAT of *Adipo-Zfp238KO* mice.

In humans, high levels of brown and beige adipocyte activity correlate with leanness, suggesting an important natural role for brown and beige adipocytes in human metabolism (Cypess et al., 2009, Saito et al., 2009, van Marken Lichtenbelt et al., 2009). Therefore, for an effective strategy to treat metabolic diseases, it is important to understand the molecular mechanism of functional regulation of the amount and/or activity of brown and beige adipocytes. Data presented here demonstrate that Zfp238, which is a co-repressor of Foxo1, likely plays a role as a metabolic regulator that can induce beiging with the potential capacity to counteract obesity and insulin resistance. Therefore, both Zfp238 and Foxo1 in adipocytes should be molecular targets for the prevention and treatment of obesity.

### Limitations of the Study

In the present study, we demonstrated that Zfp238 is a Foxo1 co-repressor and that Zfp238 in adipose tissue regulates the thermogenic program in cooperation with Foxo1. However, further analyses are required to elucidate the mechanisms by which Zfp238 inhibits Foxo1 activity and both Zfp238 and Foxo1 regulate the thermogenic program in adipocytes.

## Methods

All methods can be found in the accompanying Transparent Methods supplemental file.

## Supplemental Information

Document S1. Transparent Methods and Figures S1–S4
