# Supplementary material for: Zfp238 Regulates the Thermogenic Program in Cooperation with Foxo1
Source: iScience. 2019 Jan 7;12:87–101. doi: 10.1016/j.isci.2019.01.005 (PMC6352565; doi:10.1016/j.isci.2019.01.005)
Supplement: Document S1. Transparent Methods and Figures S1–S4 [file mmc1.pdf]

ISCI, Volume *12*

Supplemental Information

Zfp238 Regulates the Thermogenic Program in Cooperation with Foxo1

Motoko Kita, Jun Nakae, Yoshinaga Kawano, Hiroshi Asahara, Hiroshi Takemori, Haruo Okado, and Hiroshi Itoh

Transparent Methods

Antibodies and Cell Cultures

We purchased anti-FLAG (M2) and anti-tubulin from Sigma, anti-cMyc (9E10) from Santa Cruz Biotechnology Inc, anti-ZNF238 (ab67560) mouse polyclonal and anti-ZNF238 (ab118471) rabbit polyclonal from Abcam^{R}, anti-FOXO1 (L27) polyclonal antibodies from Cell Signaling Technology, and anti-FOXO1A antibody (ab12161) from Abcam^{R}. HEK293, 3T3-L1, and T37i cells were cultured as describesd previously (Nakae et al., 1999) (Nakae et al., 2008) (Sakaue et al., 1998). Staining 3T3-L1 cells with Oil Red O has been described previously (Nakae et al., 2003). For histological analysis, we used anti-CD68 (Dako Denmark A/S) and anti-UCP1 antibodies (Santa Cruz Biotechnology Inc).

Available Mice

Conditional *Zfp238KO* (Ohtaka-Maruyama et al., 2013) and *Adiponectin-Cre* (Eguchi et al., 2011) mice have been previously described elsewhere.

Animal Studies, Analytical Procedures, and Intraperitoneal Glucose and Insulin Tolerance Tests

For the following experiments, we used only male mice because they are more susceptible to insulin resistance and diabetes. Mice were fed a standard chow diet and water ad libitum, and housed in a barrier animal facility at 22–24°C with a 12-h light/dark cycle. The IPGTT, ITT, and insulin measurements were performed at 20 to 22 weeks of age as described previously (Kawano et al., 2016). All assays were performed in duplicate, and each value represents the mean of two independent determinations. The rectal temperature of mice was measured at 16 weeks of age using Thermal Sensor^{R} (Shibaura Electronics Co., Ltd). The studies of gene expression at room temperature, measurements of tissue weights, and of adipocyte size were performed at 20--24 weeks of age. Experiments about energy expenditure were performed at 14 to 16 weeks of age. All experimental protocols using mice were approved by the animal ethics committees of the Keio University School of Medicine and the International University of Health

and Welfare School of Medicine.

Measurement of Oxygen Consumption.

Mice aged 14 to 16 weeks under NCD were monitored individually in a metabolic cage (ARCO-2000; ARCO SYSTEM Inc., Kashiwa, Japan.) with free access to NCD and drinking water for 72 h. Each cage was monitored for oxygen consumption at 5-min intervals for 72 h, with the first day allowing the mice to acclimate to the cage environment. Total oxygen consumption was calculated as accumulated oxygen uptake for each mouse divided by its body weight. We measured oxygen consumption of 8 mice in each genotype. Representative graphs were drawn from mean $\pm$ SEM values calculated from data obtained in each measurement.

Cold Exposure and CL316243 Treatment

For experiments at cold exposure, 16-week-old mice were placed at 4°C for 48 h. For the stimulation with CL316243, mice were injected with CL316243 at a dose of 1mg/kg intraperitoneally for consecutive 5 days.

Immunohistochemistry, Immunofluorescence and Histological Analysis

For histological analysis, we removed the WAT from 20- to 24-week-old mice, fixed the specimens in 4% paraformaldehyde and embedded them in paraffin. We mounted consecutive 10μm sections on slides. After rehydration and permeabilization, we stained the specimens with hematoxylin and eosin. Immunofluorescence was performed as described previously (Kawano et al., 2012) using anti-UCP1antibody. After a wash with phosphate-buffered saline, the sections were sequentially incubated with secondary antibody and visualized using the Liquid DAB Substrate Chromogen System (DakoCytomation). The size and number of adipocytes in WAT were determined using a fluorescence microscope (BZ-8000, 9000, KEYENCE) by manually tracing at least more than 1000 adipocytes for each genotype (n=8-10). Measurement of number of CLSs was performed at 20 to 24 weeks of age as described previously (Fujisaka et al., 2009).

Immunofluorescence using HEK293 cells was performed as described previously (Nakae et al., 2006). After transfection with pCMV5/cMyc-WT FoxO1 and p3xFlag-CMV-Zfp238, cMyc-tagged Foxo1 was visualized in HEK293 cells with anti-FOXO1A antibody (ab12161, Abcam ^{R}, Cambridge, UK), and Alexa Fluor^{R} 488 goat anti-rabbit IgG (Invitrogen, Carlsbad, CA). FLAG-tagged Zfp238 was visualized with anti-FLAG (M2) antibody and Alexa Fluor^{R} 555 goat anti-mouse IgG (Invitrogen, Carlsbad, CA).

Construction of Expression Vectors

Constructions of pCMV5/cMyc-WT FoxO1, pCMV5/cMyc-CN FoxO1, and p3xFlag-CMV-Zfp238 has been described previously (Nakae et al., 1999) (Kawano et al., 2012) (Yokoyama et al., 2009).

Luciferase Assay

Construction of GAL4-Foxo1 expression vectors (PM-WTFoxo1) was described previously (Nakae et al., 2012). The 5XGAL4-luciferase reporter plasmid (pTAL-5XGAL4) was described previously (Katoh et al., 2006). For the 5XGAL4-luciferase assay, HEK293 cells were plated onto 12-well dishes. When the cells showed 70% to 80% confluence, transfections were carried out using 1.5μg of pTAL-5XGAL4, 0.3μg of several kinds of pM vector, and 0.6μg of p3xFlag-CMV empty vector or p3xFlag-CMV-Zfp238 expression vectors. The synthetic Renilla luciferase reporter vector (phRL-SV40; Promega) (10ng) was used as an internal control for transfection efficiency.

Construction of Knockdown Vectors of Zfp238

For knockdown of ZFP238 in HEK293 cells, we used RNAi-Ready pSIREN-RetroQ vector (Knockout RNAi Systems; Clontech Laboratories, Inc.), with 5'-GCTTCAAGTTGTTCGGATAAA-3', 5'-GCACTAATGACTATGACATGG-3', and 5'-GGAAGACTTTCTCCTGCATGT as the targeted sequences of shRNA-1, -6, and -10, respectively. We used shRNA-6 according to the results of preliminary experiments, including

the effects of Zfp238 knockdown on the 5XGAL4-luciferase reporter activity induced by PM-WTFoxo1 (data not shown). The sequence of shRNA-6 was 5'-GATCCG GCACTAATGACTATGACATGG TTCAAGAGA CCATGTCATAGTCATTAGTGCC TTTTTT TCTAGA G-3'. We selected an RNAi target sequence for Zfp238 using the Block-iT RNAi Designer (Invitrogen).

Construction of Double-knockdown Vector of both Zfp238 and Foxo1

For double knockdown of Zfp238 and Foxo1 in 3T3-L1 cells, we used the pSINsi-DK II vector (pSINsi-DK II DNA Set; TAKARA BIO Inc.), with 5'-GCACTAATGACTATGACATGG-3' and 5'-GCACCGACTTTATGAGCAACC-3' (Matsumoto et al., 2006) as the targeted sequences of Zfp238 and Foxo1, respectively. The sequences of the DNA cassette of Zfp238 knockdown, Foxo1 knockdown, Zfp238 scramble (SCR), and Foxo1 SCR were 5'-GATCC GCACTAATGACTATGACATGG CTGTGAAGCCACAGATGGG CCATGTCATAGTCATTAGTGC TTTTTT AT-3', 5'-CTAGA GCACCGACTTTATGAGCAACC GTGTGCTGTCCGGTTGCTCATAAAGTCGGTGC TTTTTT CCTGCA-3', 5'-GATCC GCGCGCGCTAGCGGGCCCGGC CTGTGAAGCCACAGATGGG GCCGGGCCCGCTAGCGCGCGC TTTTTT AT-3', and 5'-CTAGA GCGCCGGCCTTACGGGCCCCC GTGTGCTGTCCGGGGGCCCGTAAGGCCGGCGC TTTTTT CCTGCA-3', respectively.

Construction of Retroviral Vectors and Retroviral Transduction

To construct a retroviral vector encoding FLAG tagged Zfp238, we amplified the FLAG-Zfp238 cDNA fragment using the p3xFlag-CMV-Zfp238 expression vector as a template and the following primers: 5'- GGGG ACCGGT (AgeI) ATGGACTACAAAGACCATGACGGTG-3' (sense) and 5'-GGGG GGATCC (BamHI) TTATTTCCAAAGTTCTTGAGAGCTA-3' (antisense). After treatment with AgeI and BamHI, the PCR fragment was subcloned into AgeI and BamHI-treated pQCXIP Retroviral vector (Clontech). After confirming the vector sequences, we confirmed protein expression in HEK293 cells by transient transfection.

Recombinant pQCXIP, pSIREN-RetroQ, or pSINsi-DK II viral packaging was achieved by co-transfection of the plasmid with pVSV-G, which encoded viral envelopes, into GP2-293 packaging cells using Lipofectamine™ 2000 (Invitrogen). Viral supernatants were collected after 48 h. Viral supernatants were supplemented with 8 μg/ml polybrene and added to cells for infections for 24 h. Cells were selected with 2 μg/ml puromycin in case of pQCXIP and pSIREN-RetroQ or with 1500 μg/ml G418 in case of pSINsi-DK II vector, expanded, and seeded for differentiation experiments.

Isolation of Adipocytes and Stromal Vascular Fractions

The epididymal fat was removed, transferred to a 50ml tube containing KRHAG buffer (1M KCl, 1M $CaCl_2$, 1M KH_2PO_4, 1M $MgSO_4$, 5% bovine serum albumin, 200 mM HEPES (pH 7.8), 200 mM glucose), and cut into small pieces. The pieces were incubated with Collagenase type I (Wako) in KRHAG buffer (3.0mg/1.5ml) for 45 min at 37°C with gentle shaking. After filtering through 250μm nylon, the elution was centrifuged at 1500rpm for 5min at 4°C and the upper white layer as an adipocyte fraction was picked up and collected gently by pipetting. Cell pellets after centrifugation were washed twice in Pharm Lyse (BD Bioscience) buffer. After hemolytic incubation with lysing solution (BD Bioscience), the cells were washed twice again and collected as the stromal vascular fraction.

RNA Isolation and Real-time PCR

Isolation of total RNA was performed using the SV Total RNA Isolation System (Promega) according to the manufacturer's protocol. We performed reverse transcription using the PrimeScript™ RT Reagent Kit, and real-time PCR using the SYBR GREEN detection protocol by STRATAGENE (An Agilent Technologies Division, Germany). All primer sequences are available upon request.

Western Blotting

For western blotting, we homogenized tissues and lysed cells as described previously (Nakae et al., 2012). After centrifugation to remove insoluble material, the proteins in 50 µg of lysate were separated using 8% SDS-PAGE for detection of Zfp238, Foxo1, and Tubulin, or 14% SDS-PAGE for detection of Ucp1, and immunized using the indicated antibodies.

ChIP Assay

3T3-L1 cells transduced with retrovirus encoding Flag-tagged Zfp238 were seeded onto 15-cm culture dishes and induced to differentiate into mature adipocytes as described previously (Sakaue et al., 1998). After differentiation, cells were fixed with 1% formaldehyde for 1 h at 37°C. The DNA solution for Chip PCR was prepared according to the protocol in the Chip Assay Kit (Upstate). We performed immunoprecipitation with anti-FLAG (M2) antibody. We subjected the samples to PCR using primers for the enhancer, the TSS, and a region 5kb downstream from the TSS of the Ucp1 gene as described elsewhere (Iida et al., 2015). Measurement of the amounts of PCR products was performed by real-time PCR.

Statistical Analysis

We calculated descriptive statistics using one-way or two-way ANOVA with Fisher's test. All data are expressed as mean $\pm$ standard error (SEM). Significance was set at $p<0.05$.

SUPPLEMENTAL REFERENCES

Eguchi, J., Wang, X., Yu, S., Kershaw, E.E., Chiu, P.C., Dushay, J., Estall, J.L., Klein, U., Maratos-Flier, E., and Rosen, E.D. (2011). Transcriptional control of adipose lipid handling by IRF4. Cell Metab *13*, 249-259.

Fujisaka, S., Usui, I., Bukhari, A., Ikutani, M., Oya, T., Kanatani, Y., Tsuneyama, K., Nagai, Y., Takatsu, K., Urakaze, M., et al. (2009). Regulatory mechanisms for adipose tissue M1 and M2 macrophages in diet-induced obese mice. Diabetes *58*, 2574-2582.

Iida, S., Chen, W., Nakadai, T., Ohkuma, Y., and Roeder, R.G. (2015). PRDM16 enhances nuclear receptor-dependent transcription of the brown fat-specific Ucp1 gene through

interactions with Mediator subunit MED1. Genes Dev *29*, 308-321.

Katoh, Y., Takemori, H., Lin, X.Z., Tamura, M., Muraoka, M., Satoh, T., Tsuchiya, Y., Min, L., Doi, J., Miyauchi, A., et al. (2006). Silencing the constitutive active transcription factor CREB by the LKB1-SIK signaling cascade. Febs J *273*, 2730-2748.

Kawano, Y., Nakae, J., Watanabe, N., Fujisaka, S., Iskandar, K., Sekioka, R., Hayashi, Y., Tobe, K., Kasuga, M., Noda, T., et al. (2012). Loss of Pdk1-Foxo1 signaling in myeloid cells predisposes to adipose tissue inflammation and insulin resistance. Diabetes *61*, 1935-1948.

Kawano, Y., Nakae, J., Watanabe, N., Kikuchi, T., Tateya, S., Tamori, Y., Kaneko, M., Abe, T., Onodera, M., and Itoh, H. (2016). Colonic Pro-inflammatory Macrophages Cause Insulin Resistance in an Intestinal Ccl2/Ccr2-Dependent Manner. Cell Metab *24*, 295-310.

Matsumoto, M., Han, S., Kitamura, T., and Accili, D. (2006). Dual role of transcription factor FoxO1 in controlling hepatic insulin sensitivity and lipid metabolism. J Clin Invest *116*, 2464-2472.

Nakae, J., Cao, Y., Daitoku, H., Fukamizu, A., Ogawa, W., Yano, Y., and Hayashi, Y. (2006). The LXXLL motif of murine forkhead transcription factor FoxO1 mediates Sirt1-dependent transcriptional activity. J Clin Invest *116*, 2473-2483.

Nakae, J., Cao, Y., Hakuno, F., Takemori, H., Kawano, Y., Sekioka, R., Abe, T., Kiyonari, H., Tanaka, T., Sakai, J., et al. (2012). Novel repressor regulates insulin sensitivity through interaction with Foxo1. EMBO J *31*, 2275-2295.

Nakae, J., Cao, Y., Oki, M., Orba, Y., Sawa, H., Kiyonari, H., Iskandar, K., Suga, K., Lombes, M., and Hayashi, Y. (2008). Forkhead transcription factor FoxO1 in adipose tissue regulates energy storage and expenditure. Diabetes *57*, 563-576.

Nakae, J., Kitamura, T., Kitamura, Y., Biggs, W.H., 3rd, Arden, K.C., and Accili, D. (2003). The forkhead transcription factor Foxo1 regulates adipocyte differentiation. Dev Cell *4*, 119-129.

Nakae, J., Park, B.C., and Accili, D. (1999). Insulin stimulates phosphorylation of the forkhead transcription factor FKHR on serine 253 through a Wortmannin-sensitive pathway. J Biol Chem *274*, 15982-15985.

Ohtaka-Maruyama, C., Hirai, S., Miwa, A., Heng, J.I., Shitara, H., Ishii, R., Taya, C., Kawano, H., Kasai, M., Nakajima, K., et al. (2013). RP58 regulates the multipolar-bipolar transition of newborn

neurons in the developing cerebral cortex. Cell Rep *3*, 458-471.

Sakaue, H., Ogawa, W., Matsumoto, M., Kuroda, S., Takata, M., Sugimoto, T., Spiegelman, B.M., and Kasuga, M. (1998). Posttranscriptional control of adipocyte differentiation through activation of phosphoinositide 3-kinase. J Biol Chem *273*, 28945-28952.

Yokoyama, S., Ito, Y., Ueno-Kudoh, H., Shimizu, H., Uchibe, K., Albini, S., Mitsuoka, K., Miyaki, S., Kiso, M., Nagai, A., et al. (2009). A systems approach reveals that the myogenesis genome network is regulated by the transcriptional repressor RP58. Dev Cell *17*, 836-848.

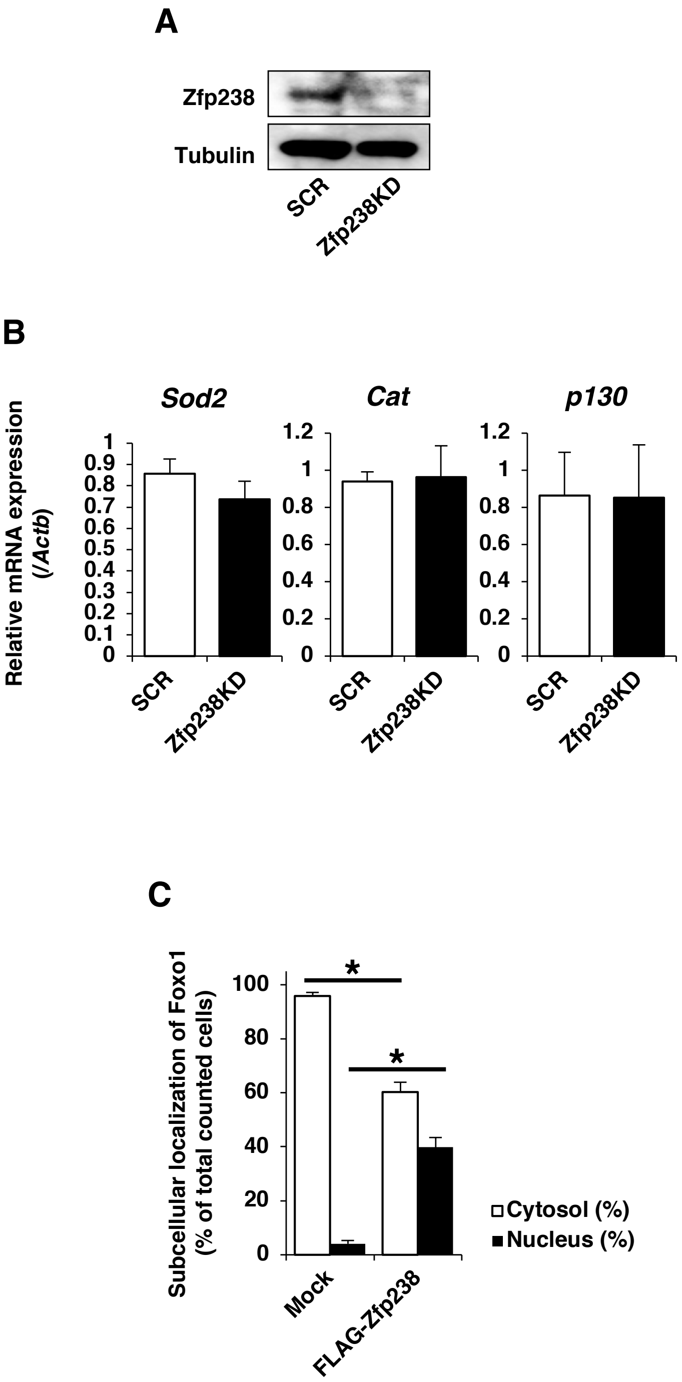
A
Zfp238
Tubulin
SCR
Zfp238KD
B
Relative mRNA expression (/Actb)
Sod2
Cat
p130
1
0.9
0.8
0.7
0.6
0.5
0.4
0.3
0.2
0.1
0
1.2
1
0.8
0.6
0.4
0.2
0
SCR
Zfp238KD
C
Subcellular localization of Foxo1 (% of total counted cells)
100
80
60
40
20
0
*
Cytosol (%)
Nucleus (%)
Mock
FLAG-Zfp238

Figure S1

Figure S1. Related to Figure 1. Generation of 3T3-L1 cells infected with the retrovirus encoding shRNA-6 Zfp238.

(A) Representative western blotting of Zfp238 in differentiated 3T3-L1 cells infected with retroviruses encoding shRNA-SCR or –Zfp238.

(B) Knockdown of Zfp238 does not induce expression of Foxo1-targeted genes in differentiated 3T3-L1 cells in the presence of serum.

Data represent the mean $\pm$ SEM from 3 independent experiments.

(C) Subcellular localization of cMyc-tagged Foxo1 in HEK 293 cells transfected with mock or FLAG-tagged Zfp238 in the presence of serum. Data represent the mean $\pm$ SEM from 3 independent experiments.
 *$p<0.05$ by one-way ANOVA.

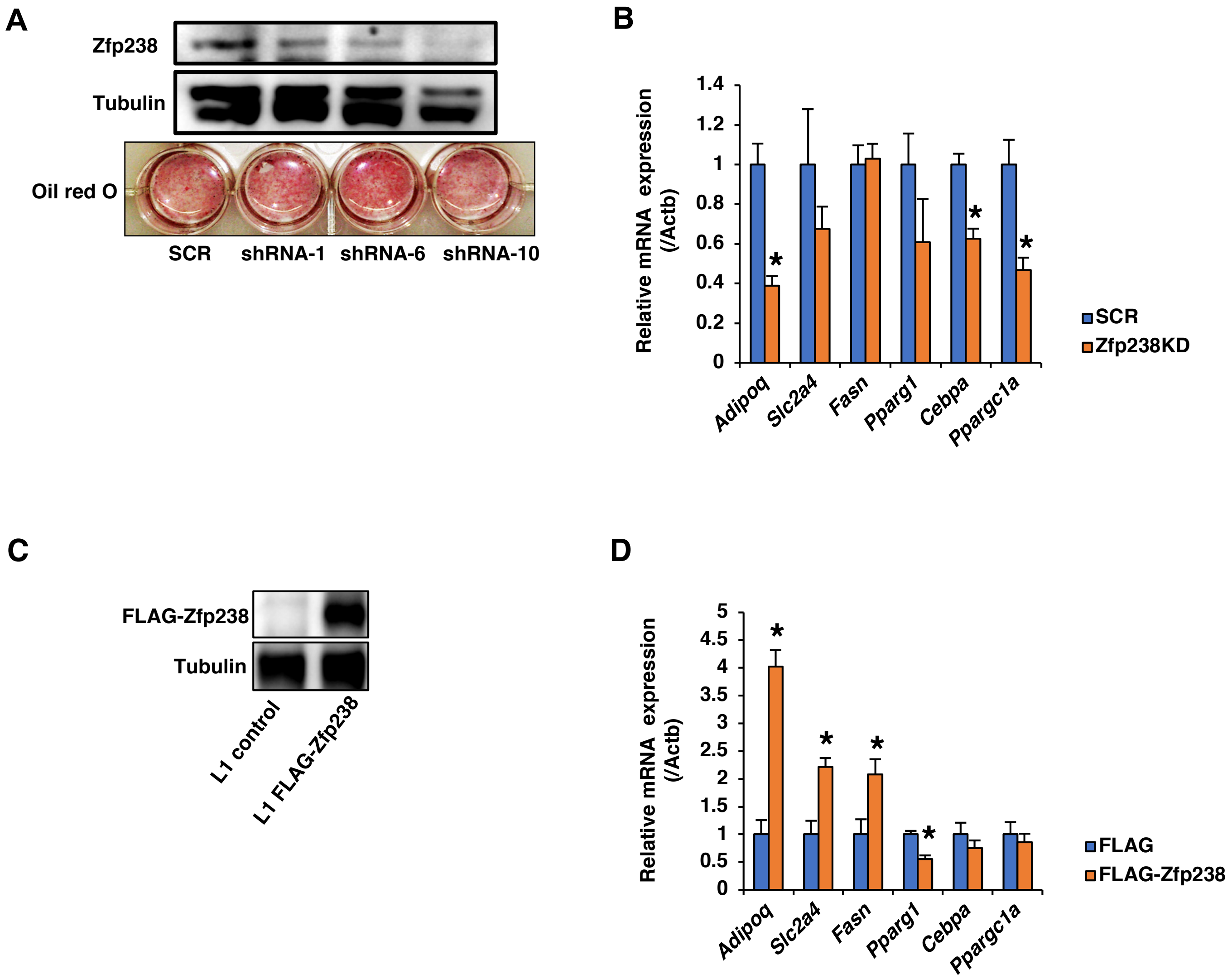
A
Zfp238
Tubulin
Oil red O
SCR
shRNA-1
shRNA-6
shRNA-10
B
Relative mRNA expression (/Actb)
1.4
1.2
1
0.8
0.6
0.4
0.2
0
Adipoq
Slc2a4
Fasn
Pparg1
Cebpa
Ppargc1a
SCR
Zfp238KD
C
FLAG-Zfp238
Tubulin
L1 control
L1 FLAG-Zfp238
D
Relative mRNA expression (/Actb)
5
4.5
4
3.5
3
2.5
2
1.5
1
0.5
0
Adipoq
Slc2a4
Fasn
Pparg1
Cebpa
Ppargc1a
FLAG
FLAG-Zfp238

Figure S2

Figure S2. Related to Figure 2. Effects of Zfp238 in 3T3-L1 cells.
(A) Representative western blotting of endogenous Zfp238 in 3T3-L1 cells infected with retroviruses encoding shRNA-SCR, shRNA-1, -6, or -10 Zfp238 at day 14 after induction of differentiation. Oil Red O staining was performed as described in EXPERIMENTAL PROCEDURES.
(B) Normalized gene expression of adipocyte-related genes in differentiated 3T3-L1 cells infected with retroviruses encoding shRNA-SCR or shRNA-Zfp238. Data are represented as the ratio of cells infected with retrovirus encoding shRNA-SCR and means ± SEM.
*$p<0.05$ by one-way ANOVA.
(C) Representative western blotting of FLAG-Zfp238 in differentiated 3T3-L1 cells infected with retrovirus encoding FLAG-Zfp238.
(D) Normalized gene expression of adipocyte-related genes in differentiated 3T3-L1 cells infected with retroviruses encoding FLAG-empty or FLAG-Zfp238 vector. Data are represented as the ratio of cells infected with retrovirus encoding FLAG-empty and means ± SEM. Experiments were performed three times. *$p<0.05$ by one-way ANOVA.

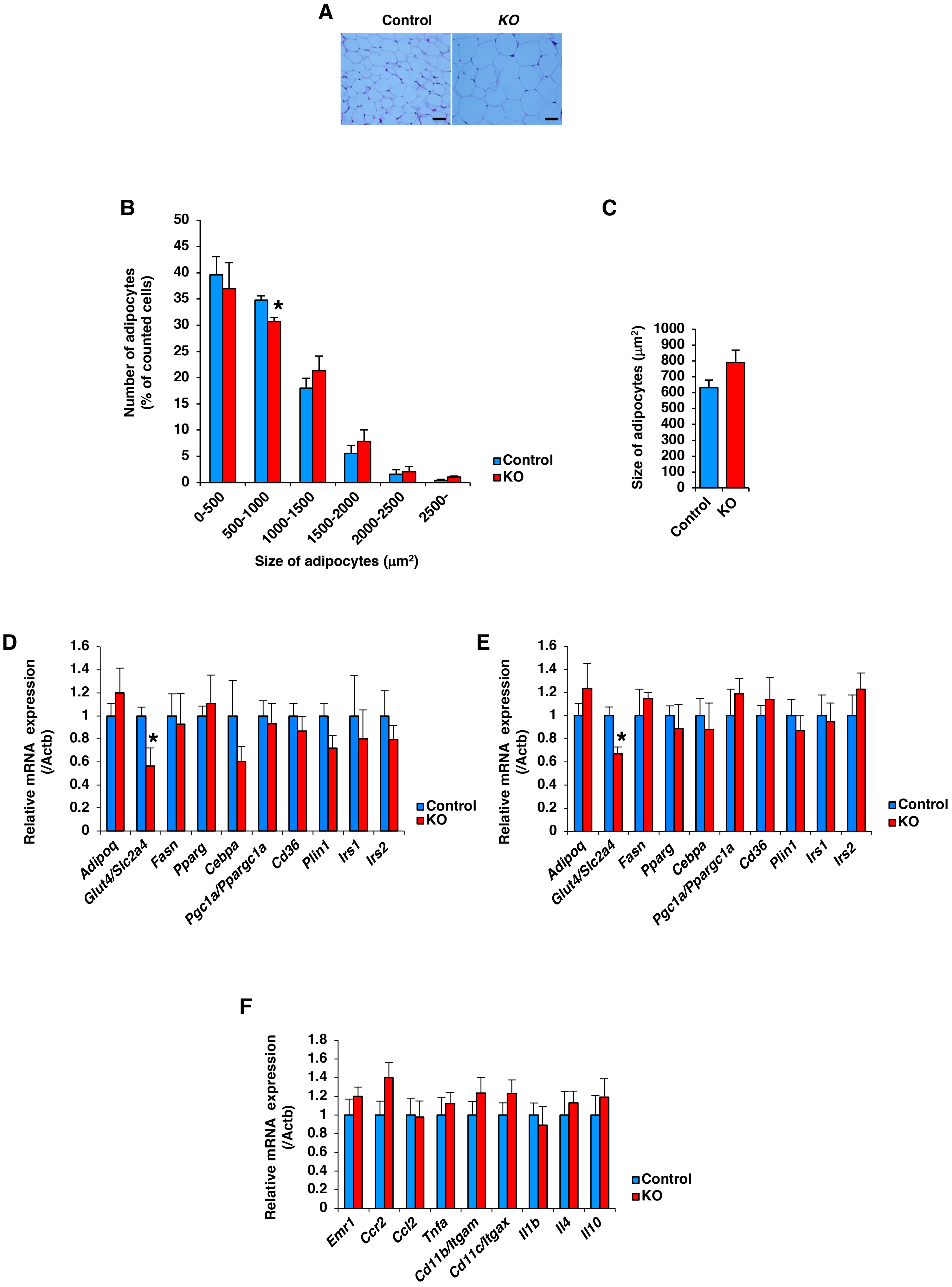

A
Control
KO
B
Number of adipocytes (% of counted cells)
50
45
40
35
30
25
20
15
10
5
0
*
0-500
500-1000
1000-1500
1500-2000
2000-2500
2500-
Size of adipocytes (μm²)
Control
KO
C
Size of adipocytes (μm²)
1000
900
800
700
600
500
400
300
200
100
0
Control
KO
D
Relative mRNA expression (/Actb)
1.6
1.4
1.2
1
0.8
0.6
0.4
0.2
0
*
Adipoq
Glut4/Slc2a4
Fasn
Pparg
Cebpa
Pgc1a/Ppargc1a
Cd36
Plin1
Irs1
Irs2
Control
KO
E
Relative mRNA expression (/Actb)
1.6
1.4
1.2
1
0.8
0.6
0.4
0.2
0
*
Adipoq
Glut4/Slc2a4
Fasn
Pparg
Cebpa
Pgc1a/Ppargc1a
Cd36
Plin1
Irs1
Irs2
Control
KO
F
Relative mRNA expression (/Actb)
1.8
1.6
1.4
1.2
1
0.8
0.6
0.4
0.2
0
Emr1
Ccr2
Ccl2
Tnfa
Cd11b/Itgam
Cd11c/Itgax
Il1b
Il4
Il10
Control
KO

Figure S3

Figure S3. Related to Figure 3. Effects of *Zfp238* Knockout in Adipose Tissue.
(A) Representative images from H&E staining of a section of epididymal fat from a 20-week-old control and *Adipo-Zfp238 KO* animals (scale bar, 20mm).
(B) Histogram of adipocyte size and number of subcutaneous fat from control (blue bar) and *Adipo-Zfp238 KO* (red bar) mice fed with NCD at the age of 20-24 weeks (n=8-10). Data represent % of total counted cells and means $\pm$ SEM. *p<0.05 by one-way ANOVA.
(C) Mean size of adipocytes of subcutaneous fat (n=8-10). Data represent each adipocyte area (mm^2) and means $\pm$ SEM. *p<0.05 by one-way ANOVA.
(D)(E) Normalized gene expression of adipose tissue-related genes in epididymal (D) and subcutaneous fats (E) (n=4-5). Data represented as the ratio of control in each gene and means $\pm$ SEM. *p<0.05 by one-way ANOVA.
(F) Normalized gene expression of immune cell-related and cytokine genes in subcutaneous fat in mice at age of 20 weeks (n=8-10). Data are the ratio of control in each gene and means $\pm$ SEM. *p<0.05 by one-way ANOVA.

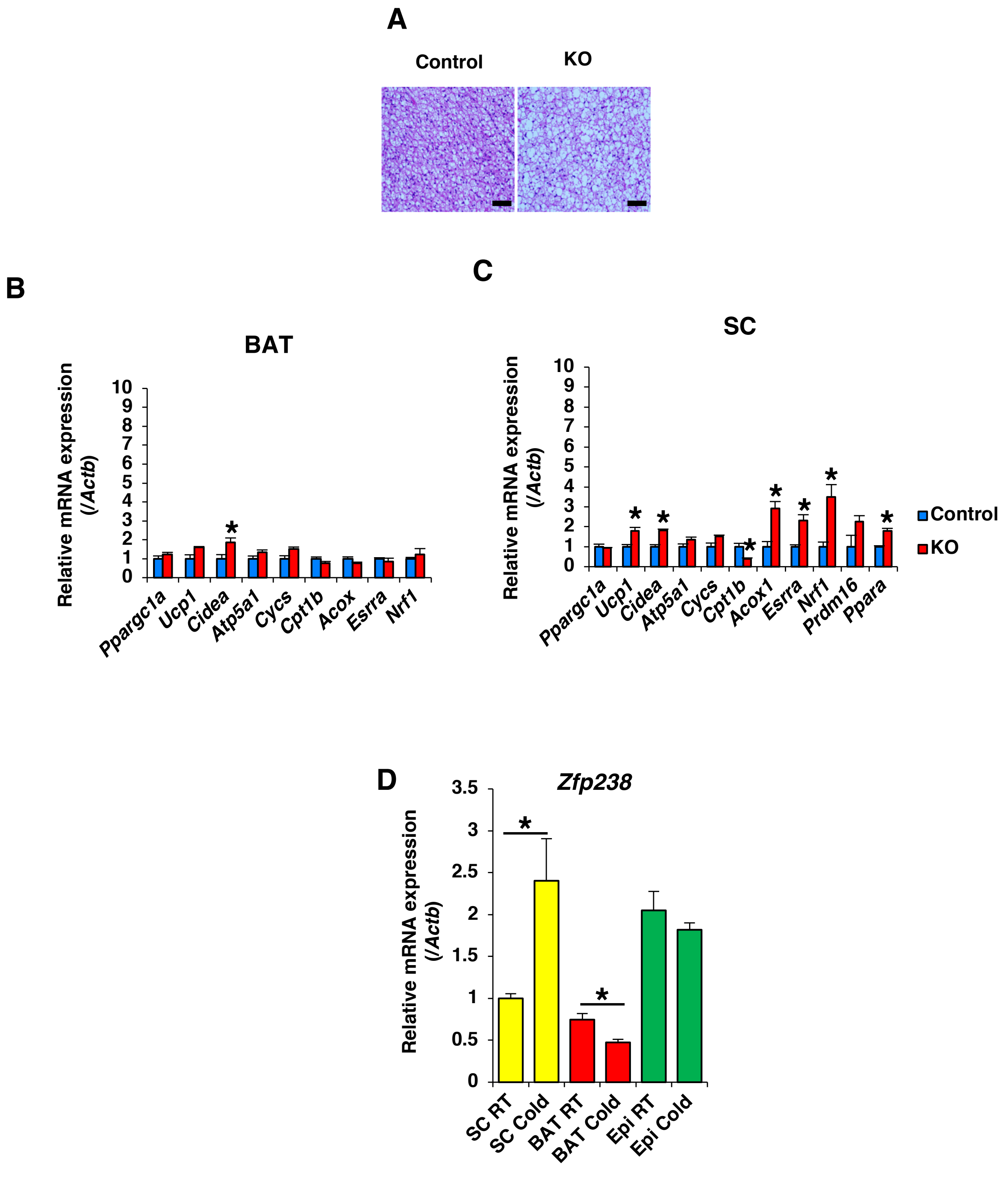
A
Control
KO
B
BAT
Relative mRNA expression (/Actb)
10
9
8
7
6
5
4
3
2
1
0
Ppargc1a
Ucp1
Cidea
Atp5a1
Cycs
Cpt1b
Acox
Esrra
Nrf1
C
SC
Relative mRNA expression (/Actb)
10
9
8
7
6
5
4
3
2
1
0
Ppargc1a
Ucp1
Cidea
Atp5a1
Cycs
Cpt1b
Acox1
Esrra
Nrf1
Prdm16
Ppara
Control
KO
D
Zfp238
Relative mRNA expression (/Actb)
3.5
3
2.5
2
1.5
1
0.5
0
SC RT
SC Cold
BAT RT
BAT Cold
Epi RT
Epi Cold

Figure S4

Figure S4. Related to Figure 4. Effects of *Zfp238* Knockout on Histology and Gene Expression of BAT and SC at Basal Ambient State.

(A) Representative images from H&E staining on section of BAT from 24-week-old control and *Adipo-Zfp238 KO* mice after cold exposure for 48 h (scale bar, 20mm).

(B) (C) Normalized gene expression of BAT- or beige adipocyte-related genes in BAT (B) or SC (C) from control and *Adipo-Zfp238 KO* mice at the age of 16 weeks (n=4-6). Data are represented as the ratio of control in each gene and means $\pm$ SEM. *$p<0.05$ by one-way ANOVA.

(D) *Zfp238* gene expression in subcutaneous adipose tissue (SC), BAT, and epididymal adipose tissue (Epi) of control mice fed with NCD at age 16 weeks at room temperature (RT) or under cold exposure (4°C, 48 h) (n=4). Data represent the ratio of RT and means $\pm$ SEM. *$p<0.05$ by one-way ANOVA.
